# In Vitro and In Silico Evaluation of the Pyrolysis of Polyethylene and Polypropylene Environmental Waste

**DOI:** 10.3390/polym17222968

**Published:** 2025-11-07

**Authors:** Joaquín Alejandro Hernández Fernández, Katherine Liset Ortiz Paternina, Jose Alfonso Prieto Palomo, Edgar Marquez, Maria Cecilia Ruiz

**Affiliations:** 1 Chemistry Program, Department of Natural and Exact Sciences, San Pablo Campus, Universidad de Cartagena, Cartagena de Indias 130015, Colombia; kortizp1@unicartagena.edu.co (K.L.O.P.); jprietop@unicartagena.edu.co (J.A.P.P.); 2 Department of Natural and Exact Science, Universidad de la Costa, Barranquilla 080002, Colombia; 3 Grupo de Investigaciones en Química Y Biología, Departamento de Química Y Biología, Facultad de Ciencias Básicas, Universidad del Norte, Carrera 51B, Km 5, Vía Puerto Colombia, Barranquilla 081007, Colombia; ebrazon@uninorte.edu.co; 4 Agencia Nacional de Hidrocarburos, Bogotá 110221, Colombia; maria.ruiz@anh.gov.co

**Keywords:** catalytic pyrolysis, polypropylene, high-density polyethylene, HMOR zeolite, Aspen Plus simulation, DFT calculations, plastic-waste valorization

## Abstract

Plastic pollution, driven by the durability and widespread use of polyolefins such as polypropylene (PP) and high-density polyethylene (HDPE), poses a formidable environmental challenge. To address this issue, we have developed an integrated multiscale framework that combines thermocatalytic experimentation, process-scale simulation, and molecular-level modeling to optimize the catalytic pyrolysis of PP and HDPE waste. Under the identified optimal conditions (300 °C, 10 wt % HMOR zeolite), liquid-oil yields of 60.8% for PP and 87.3% for HDPE were achieved, accompanied by high energy densities (44.2 MJ/kg, RON 97.5 for PP; 43.7 MJ/kg, RON 115.2 for HDPE). These values significantly surpass those typically reported for uncatalyzed pyrolysis, demonstrating the efficacy of HMOR in directing product selectivity toward valuable liquids. Above 400 °C, the process undergoes a pronounced shift toward gas generation, with gas fractions exceeding 50 wt % by 441 °C, underscoring the critical influence of temperature on product distribution. Gas-phase analysis revealed that PP-derived syngas contains primarily methane (20%) and ethylene (19.5%), whereas HDPE-derived gas features propylene (1.9%) and hydrogen (1.5%), highlighting intrinsic differences in bond-scission pathways governed by polymer architectures. Aspen Plus process simulations, calibrated against experimental data, reliably predict product distributions with deviations below 20%, offering a rapid, cost-effective tool for reactor design and scale-up. Complementary density functional theory (DFT) calculations elucidate the temperature-dependent energetics of C–C bond cleavage and radical formation, revealing that system entropy increases sharply at 500–550 °C, favoring the generation of both liquid and gaseous intermediates. By directly correlating catalyst acidity, molecular reaction mechanisms, and process-scale performance, this study fills a critical gap in plastic-waste valorization research. The resulting predictive platform enables rational design of catalysts and operating conditions for circular economy applications, paving the way for scalable, efficient recovery of fuels and chemicals from mixed polyolefin waste.

## 1. Introduction

The proper management of plastic waste represents an increasingly significant environmental challenge of global magnitude: annual global plastic production reached approximately 390 million tonnes in 2021, up from 335 million tonnes in 2016, and is projected to exceed 500 million tonnes by 2050 [1,2,3,4,5,6]. This high production, coupled with the persistent resistance of polymers to natural degradation, has led to a significant accumulation of plastic waste in the environment, adversely affecting both terrestrial and aquatic ecosystems [7,8]. Among the most widely used and therefore most abundant polymers in waste streams are polypropylene (PP) and high-density polyethylene (HDPE), materials whose widespread application in both industrial and domestic sectors significantly contributes to the total volume of solid waste generated [9,10,11]. Faced with this problem, it has become imperative to develop and implement efficient, sustainable, and technologically viable strategies that enable the valorization and recycling of plastic waste, thereby minimizing its environmental impact and promoting the rational use of resources [12,13,14,15,16]. Among the various technologies available, pyrolysis offers several advantages that make it particularly attractive for the recovery of plastic waste: it is performed in the absence of oxygen, avoiding the emission of dioxins and furans; it converts recalcitrant macromolecules into fuel oils, synthesis gases, and solid carbonaceous waste (char) with a high energy density; it tolerates heterogeneous, contaminated, or mixed streams without requiring prior exhaustive sorting; and it can be integrated into circular economy schemes through the simultaneous recovery of energy and raw materials [17,18,19]. The efficiency and selectivity of the pyrolysis process critically depend on multiple operating variables and the catalytic system used [20]. Among the most determining factors are the operating temperature, which influences the kinetics and mechanisms of degradation; the nature and concentration of the catalyst, whose design and characteristics modulate the decomposition pathway and product distribution; the catalyst-to-raw-material ratio; and the specific type of plastic material subjected to the process [21,22,23,24,25]. The synergistic interaction of these parameters defines the quality, quantity, and composition of the resulting products; therefore, their study and optimization are essential for technological scalability and efficiency.

Over the last decade, reactive molecular dynamics with the ReaxFF force field has become a pivotal tool for unveiling atomistic reaction networks in polymer pyrolysis [26,27,28]. Liu et al. [29] modelled the thermal decomposition of HDPE (>7000 atoms) and reproduced the experimentally observed evolution of gaseous products, identifying key mechanisms such as homolytic C–C cleavage and β-scission. More recently, Li et al. [30] combined large-scale ReaxFF simulations with automatic reaction-class recognition to map product formation during PP pyrolysis, from light olefins to polycyclic aromatic hydrocarbons. These and other investigations demonstrate that ReaxFF overcomes the intrinsic temporal and spatial limitations of conventional quantum-chemical or experimental approaches, delivering mechanistic, kinetic, and energetic insights that are otherwise inaccessible.

The present study combines detailed Aspen Plus and DFT modeling simulations to quantify the advantages of modified catalysts in product distribution management, rationalize at the molecular scale how operational variables govern PP and HDPE fragmentation pathways, and establish optimal conditions to maximize liquid-fuel yield while minimizing undesirable by-products. By explicitly linking atomistic mechanisms to process-scale performance, the work seeks to advance sustainable and circular strategies for handling high-volume plastic waste.

## 2. Materials and Methods

### 2.1. PP and HDPE Samples

Plastic waste, mainly post-consumer bottles and PP and HDPE fractions recovered from landfills and collection centers in Cartagena (Colombia), underwent a two-stage sorting protocol: first, macroscopic manual separation based on RIC resin codes #2 (HDPE) and #5 (PP), supplemented by criteria of color, rigidity, and shape, isolating unmarked pieces for instrumental verification; second, a densimetric separation by sinking–floating in a water–ethanol solution (ρ ≈ 0.93 g cm^−3^) in which PP (ρ = 0.90–0.91 g cm^−3^) and HDPE (ρ = 0.94–0.96 g cm^−3^) floated, while higher-density polymers and impurities sank and were discarded. After sorting, the polymers were washed with deionized water and neutral detergent, dried at 60 °C for 24 h, and cut into 2 cm × 2 cm flakes. The immediate properties (moisture, volatile matter, fixed carbon, and ash) were determined in a PerkinElmer TGA-7 thermogravimetric analyzer (PerkinElmer, Inc., Waltham, MA, USA) under a nitrogen atmosphere (50 mL min^−1^) between 25 and 800 °C at 10 °C min^−1^ using ~10 mg of sample, while the elemental composition (C, H, N, O, and S) was quantified in a Bruker elemental analyzer (Bruker Corporation, Billerica, MA, USA).

### 2.2. Catalysts

The catalysts employed in the catalytic pyrolysis of PP and HDPE were mordenite zeolite in its protonated form (H-Mordenite, abbreviated H-MOR), whose molar ratio n(Si/Al) is detailed in Table 1, and its three copper-modified derivatives: CuCl_2_-H-MOR, CuSO_4_-H-MOR, and Cu(NO_3_)_2_-H-MOR. Table 1 presents the main physicochemical properties of the starting zeolite.

The following reagents were used to modify the catalyst: Merck copper sulfate (CuSO_4_∙5H_2_O); Merck copper nitrate (CuNO_3_)_2_∙3H_2_O; Sigma-ALDRICH copper chloride (CuCl_2_); and deionized water. HMOR was introduced into different 0.1 M solutions prepared with various salts (copper sulfate, copper acetate, copper chloride, and copper nitrate). HMOR modification was started by adding 2 g of catalyst to 200 mL of the copper salt solution used in this study. The combination was stirred for a full day at room temperature, then washed, filtered, and dried. Before being used in the pyrolytic process, all catalysts were pelletized, ground, and filtered to achieve particle sizes ranging from 75 to 180 μm. The catalyst (0.2–0.3 g) was then activated by heating it in the reactor with circulating nitrogen (at a rate of 50 mL min^−1^) to a temperature of 120 °C, increasing by 60 °C per hour. After two hours, the temperature was increased to 520 °C at 120 °C per hour. After 5 h at 520 °C, the reactor was cooled to the required reaction temperature.

### 2.3. Pyrolysis and Thermodegradation Reactor

**Figure 1:**
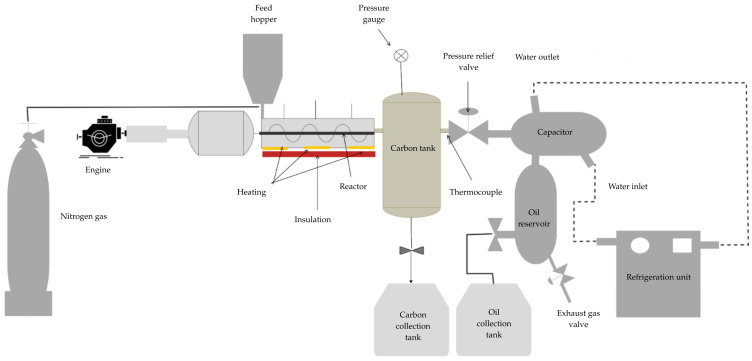
Schematic of the experimental pyrolysis setup.

This experimental stage was carried out using a quartz pyrolyzer, where the reactor was placed in a horizontal tube furnace and another furnace with precise temperature control. The tests were conducted using approximately 40 g of each PP and HDPE sample, maintaining the operating temperature within a range of 400 to 600 °C. Nitrogen with a purity of 99.9% and a flow rate of 100 mL min^−1^ was used. The experimental procedure is detailed in Figure 1. This investigation analyzed two of the fundamental variables during the pyrolysis process (catalyst quantity and reaction temperature) and their impact on the production of solid, liquid, and gaseous products. In the investigations to improve the performance, the process variables were analyzed to achieve the best possible performance. Adjustments were made to the experimental data to adapt them to the correlation, which was later reflected in graphical representations of surfaces and contours showing the ideal conditions. In the optimization investigation, PP and HDPE were used as shown in Table 2.

### 2.4. Simulation by Aspen Plus

**Figure 2:**
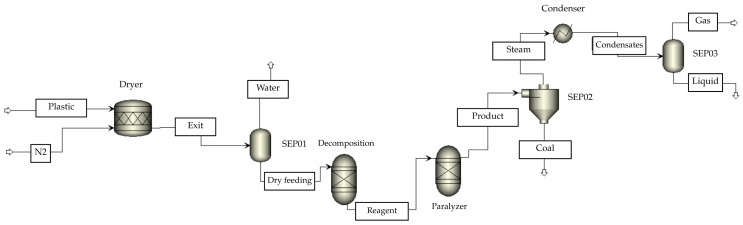
Prototype of the pyrolysis process in Aspen Plus; char (solid carbon residue).

Table 3 presents the experimental design for the thermal and catalytic pyrolysis of PP and HDPE. Based on previous studies that demonstrated high levels of performance, Aspen Plus (version 8.7) [31] was used to simulate the process under two approaches: a thermodynamic model with RGIBBS reactors and a kinetic model with RBATCH reactors. For the prediction of phase equilibria and thermodynamic properties of the gaseous and liquid streams at high temperature, the Peng-Robinson (PR-EOS) package, recognized for its reliability in hydrocarbon mixtures under pyrolysis conditions, was selected. In addition, for liquid fractions with non-ideal interactions, the NRTL model was applied to improve the accuracy of activity estimation. Solid feedstocks (waste plastics) were initially classified as non-routine streams and, using an R-YIELD reactor (DESCOMP module), were converted into routine substances for introduction into the REACTOR module. The products were divided into ash and liquid or gaseous fractions, which were separated and cooled to obtain synthetic fuels and non-condensable gases. Figure 2 illustrates the simulation scheme, and the operational details of each block are described in Table 4.

### 2.5. Computer Simulations

A computational study on the thermal decomposition of PP was performed using the B3LYP/6-311G (d,p) computational level [32]. Tests were conducted under different conditions to analyze their effect on pyrolysis. Four fundamental temperatures were considered for the evaluation: 25.0 °C, 450.0 °C, 500.0 °C, and 550.0 °C. Each of these temperatures was evaluated under three pressures: 1 atm, 10 atm, and 20 atm, to understand the behavior of PP under various temperature and pressure conditions. Particular attention was given to the HOMO and LUMO orbitals (Highest Occupied Molecular Orbital and Lowest Unoccupied Molecular Orbital, respectively), since these are essential to understand the reactivity of the produced fragments. Furthermore, electrostatic potential (EPM) maps corresponding to each relevant fragment were extracted during the pyrolysis process. These diagrams and maps were calculated for each optimized structure under the specified temperature and pressure conditions.

## 3. Results and Discussion

### 3.1. Characterization: Approximate and Final Analysis

During the proximate analysis, the mass reduction was calculated up to a temperature of 105 °C to determine the moisture content. To calculate the volatile matter (VM), the mass decrease was recorded between 105 °C and 950 °C. To quantify the ash, a procedure was carried out that involved subjecting the sample to high temperatures in a special oven, followed by evaluation of the sample’s residual weight. To determine the fixed carbon (FC), the moisture content, volatile matter (VM), and ash were subtracted from the total value of 100%. During the final analysis, the plastics were burned in a furnace at 1000 °C with oxygen, and the resulting residues were then investigated with an analytical instrument. The contents of C, H_2_, N_2_, and S in the raw material were examined. The results of the analyses carried out for HDPE and PP plastics are presented in Table 5.

When characterizing the HMOR catalyst and its salt modifications, their acidic properties are relevant. These measurements were carried out using the potentiometric technique, which is less expensive. Table 6 presents the central values resulting from the titrations performed on the catalysts, which were obtained from the potentiometric titration graphs.

The HMOR presented the highest acid strength value, namely, 425 [mV], and the highest number of acid sites (2.3 meq/g). In the case of the HMOR that was subjected to an ion exchange process with copper salts, it can be noted that its acidity and number of acid sites were lower than those reported for the unmodified HMOR. The reason for this is the substitution of protons in the zeolite by the Cu^+2^ ion, which leads to a reduction in the acidity generated by the Brönsted acid centers. In these situations, the metal ion is responsible for neutralizing a portion of the negative charges of the structure, especially those present on the outer surface of the porous material.

### 3.2. Catalytic Pyrolysis

This study evaluated the effect of temperature, catalyst type, and catalyst-to-substrate ratio on the pyrolysis of polypropylene (PP) and high-density polyethylene (HDPE) waste. Experimental results were fitted to a correlation model and plotted on graphical surfaces to identify the optimum conditions. Thermal decomposition of PP started at 245 °C, while raising the temperature to 400 °C showed a decrease in overall conversion and an increase in gas generation. The highest yield in overall conversion was achieved at 300 °C with the 10 wt.% catalyst, obtaining as the main product a liquid oil whose proportion was 60.80 wt.% (Table 7). To determine the composition of this oil, the samples were analyzed by gas chromatography coupled to mass spectrometry (GC-MS).

**Figure 3:**
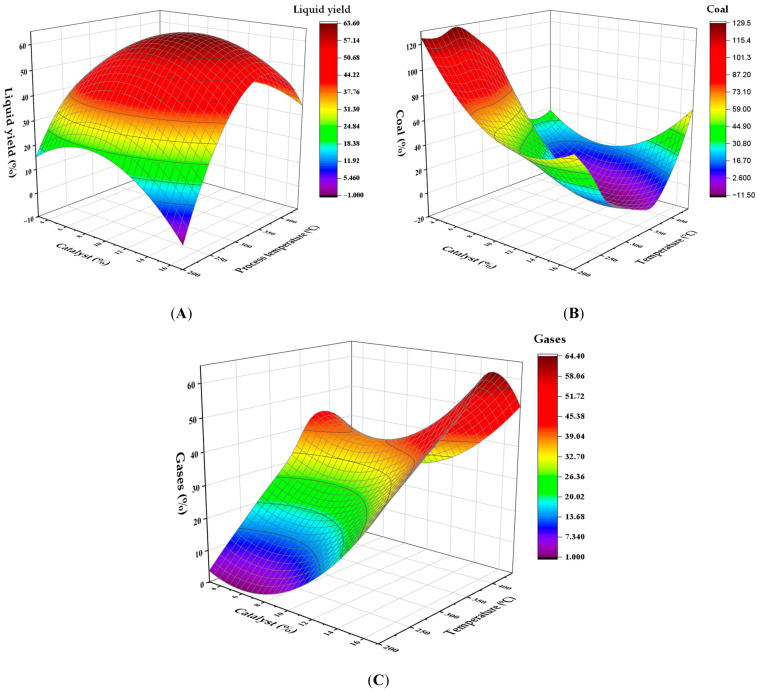
Response surface for the effect of temperature and catalyst concentration on the yield of (**A**) liquids, (**B**) solid carbonaceous residue, and (**C**) gases in PP pyrolysis.

To visualize and quantify the progress of catalytic pyrolysis of PP plastic waste, three-dimensional graphical representations (response surfaces) were designed that show the combined influence of two variables: process temperature and the amount of catalyst relative to the feedstock (% *m*/*m*), on three fundamental outcomes: liquid generation, carbon residue accumulation, and gas release. The solutions are shown in Figure 3A–C. Figure 3A shows that the maximum liquid product production is achieved at a temperature of 300 °C, using a 10% HMOR catalyst, resulting in a maximum yield of 60.8% by weight. As the temperature drops below 250 °C, the efficiency decreases, whereas above 400 °C, the liquid efficiency is also affected by the production of gases. Temperature had the most significant effect on the response variable (*p* = 0.001; F = 15.86), while the catalyst proportion was not significant (*p* = 0.932). Regarding carbon (Figure 3B), its concentration increases at temperatures below 200 °C but decreases at 300 °C, promoting the liquid phase. Under high temperatures and with catalysts above 15%, an increase in carbon residue generation is likely due to additional carbonization. Regarding gases (Figure 3C), their production increases with increasing temperature, reaching over 50% by weight at 441 °C, with a catalyst level of 10%. At lower temperatures (below 200 °C), gas production is limited, ranging only from 1% to 5%.

The same experimental methodology was implemented for HDPE. Table 8 presents the results derived from the process.

**Figure 4:**
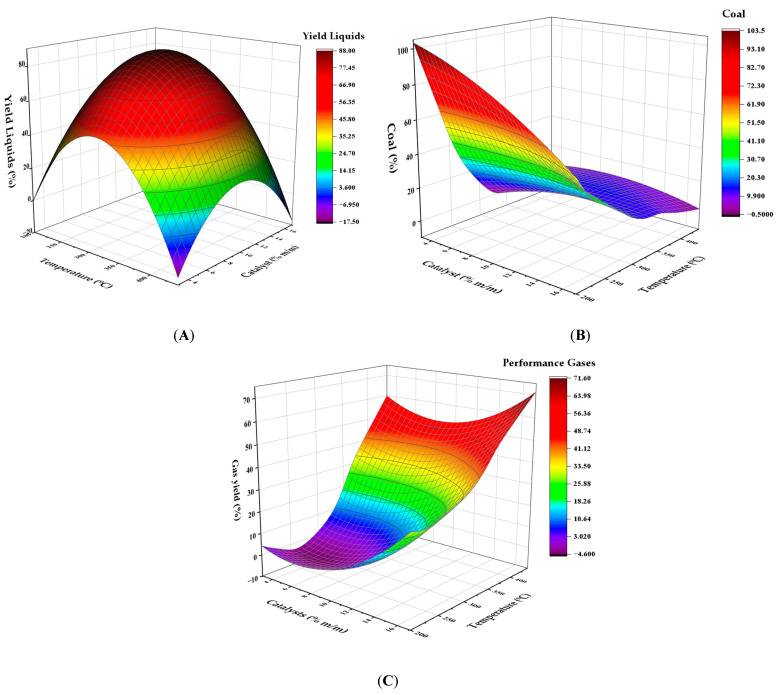
Response surface for the effect of temperature and catalyst concentration on the yield of (**A**) liquids, (**B**) solid carbonaceous residue, and (**C**) gases in the pyrolysis of HDPE.

Figure 4 show that maximum liquid production is achieved at 300 °C using the 10% catalyst. According to previous studies, moderate temperatures favor fluid formation, unlike very low temperatures (<250 °C) or very high temperatures (>400 °C), which reduce production. At temperatures below 250 °C, optimal liquid production efficiency is reduced due to partial conversion of the polymeric material (Figure 4A). On the other hand, when the temperature exceeds 400 °C, gas production is favored, which reduces the presence of liquids. Char generation (Figure 4B) was more critical at temperatures below 200 °C, where the partial decomposition of the polymer produces a greater amount of solid waste. However, when the temperature exceeds 300 °C and the catalyst content exceeds 15%, an increase in carbon concentration is observed, probably due to secondary carbonization processes. Gas generation (Figure 4C) increased significantly with increasing temperature, exceeding 50% by mass at 441 °C, indicating the complete decomposition of the polymers into light volatile substances.

### 3.3. Design Verification Using Aspen Plus

The plastic pyrolysis model was validated in Aspen Plus by comparing the simulation results (liquid and syngas generation) for PP and HDPE with experimental values, varying the pyrolysis temperature. The solid fraction, which is less than 2%, was excluded to focus on the liquid and gaseous phases. Table 9 compares the characteristics of the liquids generated by the pyrolysis of these plastics with the reference values for gasoline and diesel, as specified in Colombian Resolution 898/95. The discrepancies between the simulation results and the reference values do not exceed 20%.

The PCSs (Higher Heating Values) of the liquid fractions of both polymers are very similar: PP-L = 40.6 MJ kg^−1^ and HDPE-L = 40.2 MJ kg^−1^, so that PP only has a marginal energy advantage. In terms of octane rating, PP-L has 87.4 MON and 97.5 RON, while HDPE-L reaches 85.5 MON and 95.6 RON; therefore, PP-derived fuel actually offers slightly better performance in high-compression ratio engines.

The anti-knock index (AKI = (RON + MON)/2) is 92.5 for PP-L and 90.5 for HDPE-L, confirming that PP has greater resistance to knocking and, consequently, greater stability.

Finally, the density at 15 °C is 0.88 g cm^−3^ (PP-L) and 0.92 g cm^−3^ (HDPE-L); in this case, HDPE is slightly denser, which may imply a slight advantage in energy per unit volume.

### 3.4. Characterization of the Liquid and Gaseous Phases of PP and HDPE Pyrolysis

Table 10 presents the main liquid and gaseous products obtained from PP and HDPE pyrolysis, expressed as weight-percent averages over five replicates. In the liquid fraction from PP, 59.5% falls under ‘Others,’ while individually quantified compounds, C_12_H_10_ (10.0%), C_11_H_10_ (9.5%), C_14_H_10_ (8.1%), and C_8_H_10_ (4.3%), constitute smaller shares. For HDPE, ‘Others’ accounts for 30.1%, and the most abundant identified species are toluene (C_7_H_8_, 22.2%), C_8_H_10_ (14.3%), and tetradecane (C_14_H_30_, 12.2%). In the gas phase, PP yields primarily methane (CH_4_, 20.0%) and ethylene (C_2_H_4_, 19.5%), whereas HDPE produces CH_4_ (5.0%), C_2_H_4_ (4.6%), propylene (C_3_H_6_, 1.9%), and hydrogen (H_2_, 1.5%). These distributions highlight the distinct C–C bond-scission pathways of each polymer and are crucial for tailoring applications in energy generation or chemical feedstock production [33,34,35,36,37,38].

**Figure 5:**
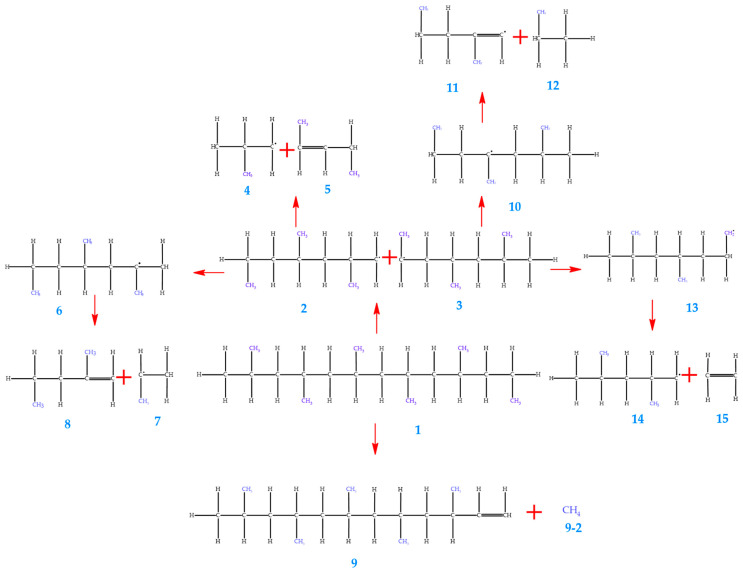
Proposed mechanism for thermal degradation of PP.

Figure 5 shows the proposed reaction mechanism for the thermal degradation of polypropylene (PP), emphasizing the formation of reactive intermediates. In this process, the cleavage of C–C bonds takes place under high temperatures, leading to the generation of gaseous and liquid products. The radical intermediates were modeled at critical temperatures of 500 °C and 550 °C to evaluate their energetic and structural behavior. The analysis of the system’s energy characteristics (E. System) in the 450–550 °C range reveals values that remain essentially unchanged (≈−3.09 × 10^−12^ J). At 450 °C and 1 atm, the energy is −3.09 × 10^−12^ J, and at 550 °C, it remains practically the same. This minimal variation indicates that, within this temperature window, the degradation process of PP does not involve significant differences in overall system stability (Table 11 and Table 12).

This behavior suggests that the apparent energy required for bond cleavage is relatively insensitive to temperature changes in this range. Instead of reflecting increased instability, the constancy of energy values may point to compensatory structural rearrangements within the polymer. Such rearrangements could favor the formation of intermediates or degradation products that require similar energetic contributions, thereby maintaining the overall stability of the system despite the increase in temperature.

Additionally, this behavior may reflect an increase in the system’s entropy, as evidenced by the rise in molecular entropy (E. Entropy) with temperature. For example, in the case of molecule 1, entropy increases from 1.25 × 10^6^ kJ/mol at 450 °C to 1.36 × 10^6^ kJ/mol at 550 °C. This growth in entropy indicates a broader energy dispersion, which can be associated with the generation of a larger number of products and with greater freedom of motion for the resulting fragments. Thus, although the system’s total energy does not decrease significantly, the process becomes thermodynamically more favorable due to the entropic contribution. An analysis of the Gibbs free energy for molecule 9 reveals that, as temperature increases, the Gibbs energy approaches zero. This observation demonstrates that pyrolysis reactions become more favorable at 550 °C. Such a result is consistent with the general trend that polymer pyrolysis at high temperatures promotes bond dissociation and the formation of smaller molecular fragments, reflected in the decrease in Gibbs free energy.

**Figure 6:**
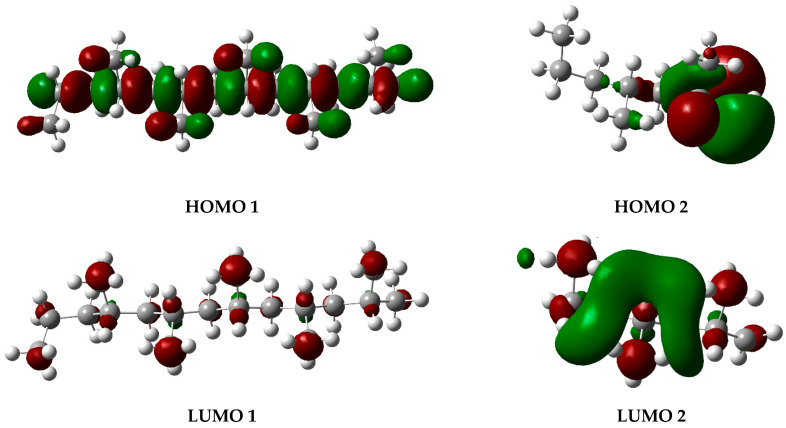

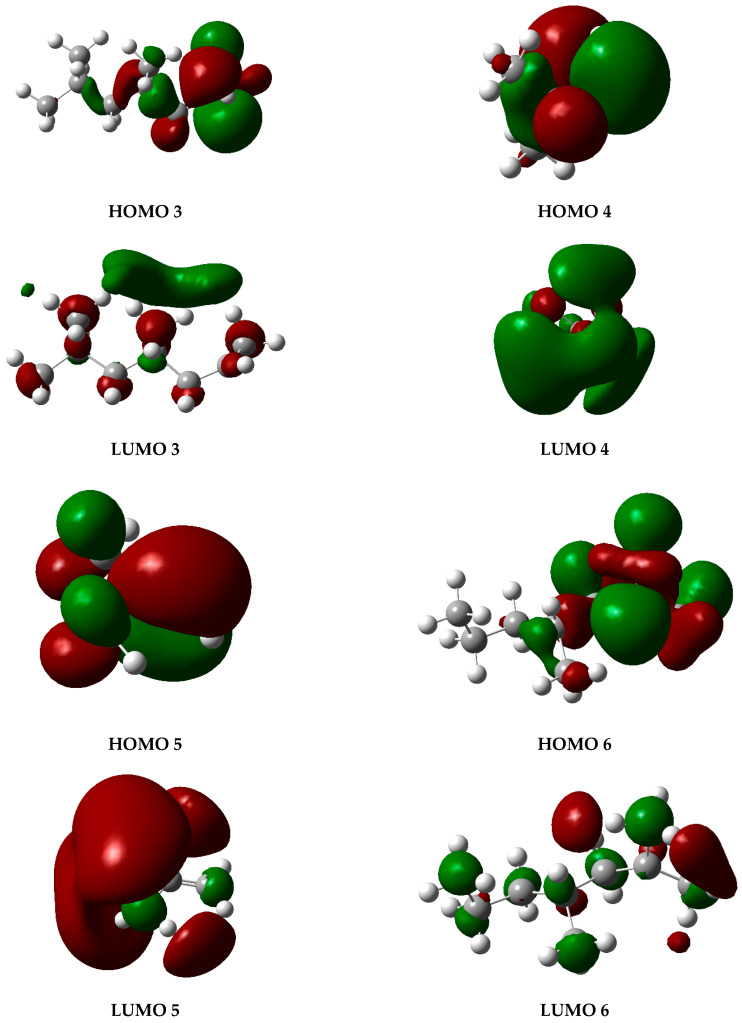

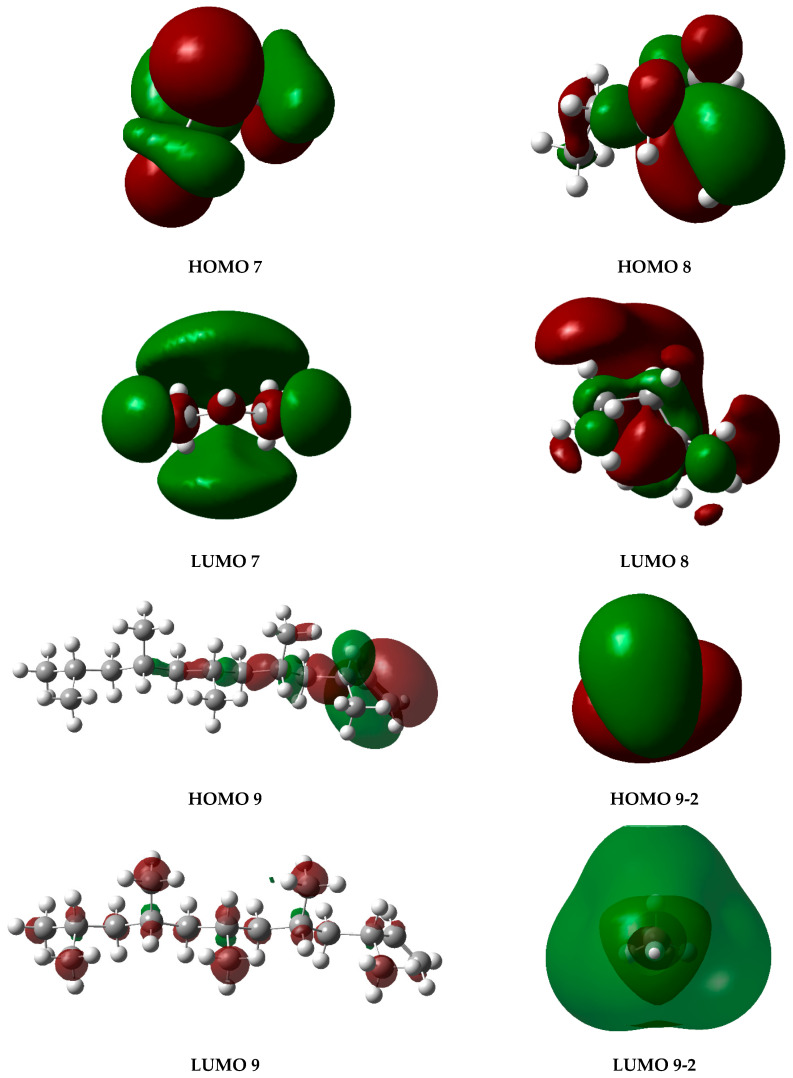

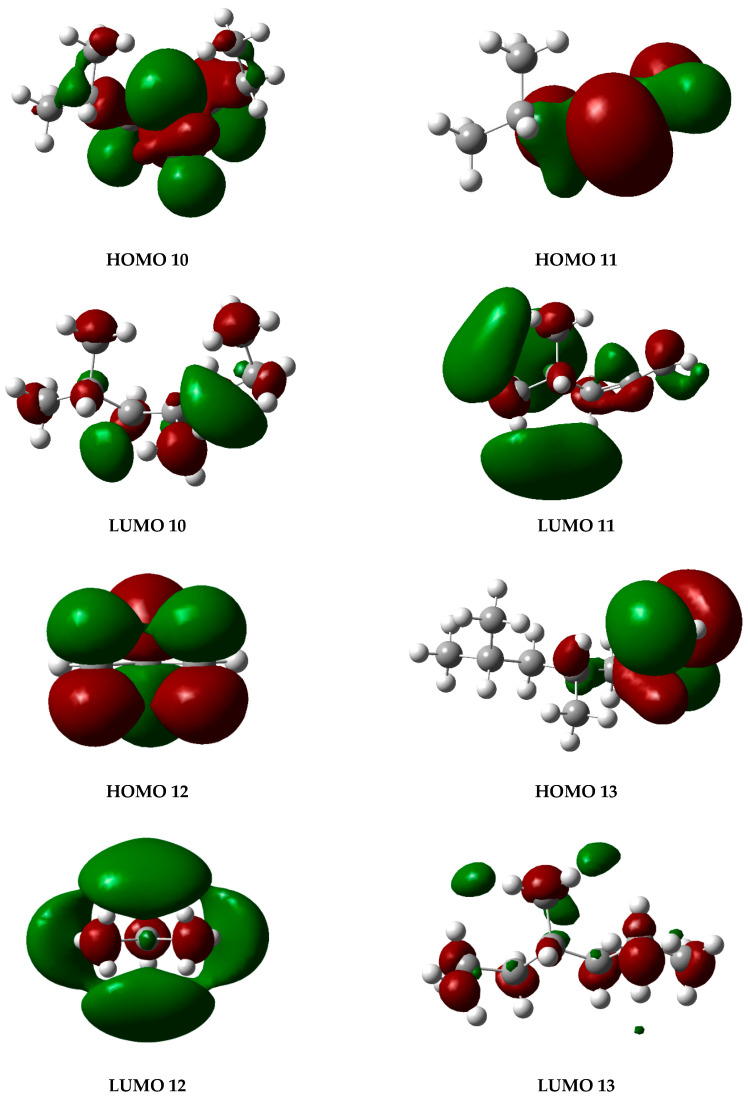

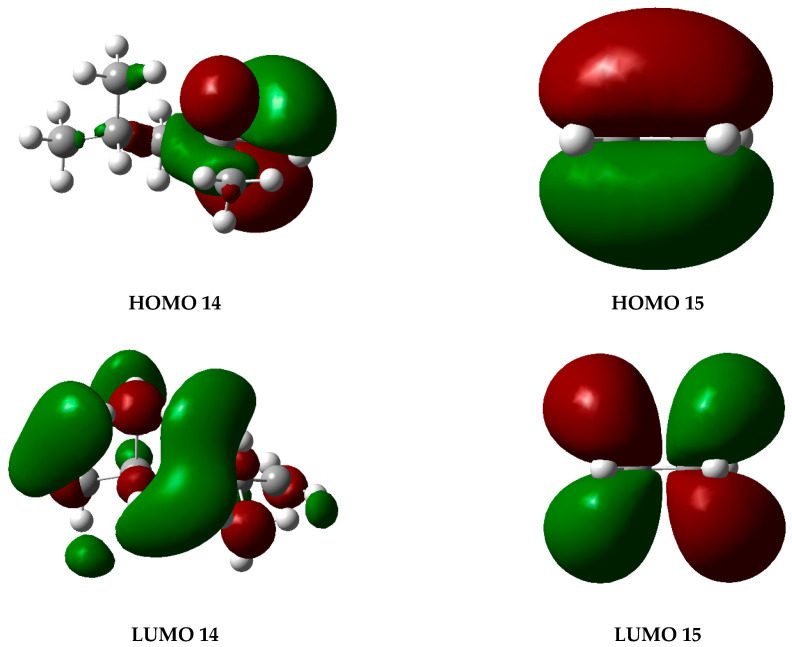
HOMO and LUMO molecular orbital diagrams of PP degradation fragments. Gray spheres represent carbon atoms and white spheres represent hydrogen atoms, while red and green lobes correspond to opposite phases of the orbital wavefunction.

The graphs presented in Figure 6 illustrate the molecular orbitals of PP at 450 °C, obtained through optimization and frequency calculations. These images depict the electron distribution in the HOMO and LUMO orbitals of the fragments generated during the pyrolysis process, as proposed in the mechanism shown in Figure 5. The HOMO represents the highest-energy orbital, containing the principal electrons, while the LUMO remains unoccupied and constitutes the optimal site for electron excitation.

**Figure 7:**
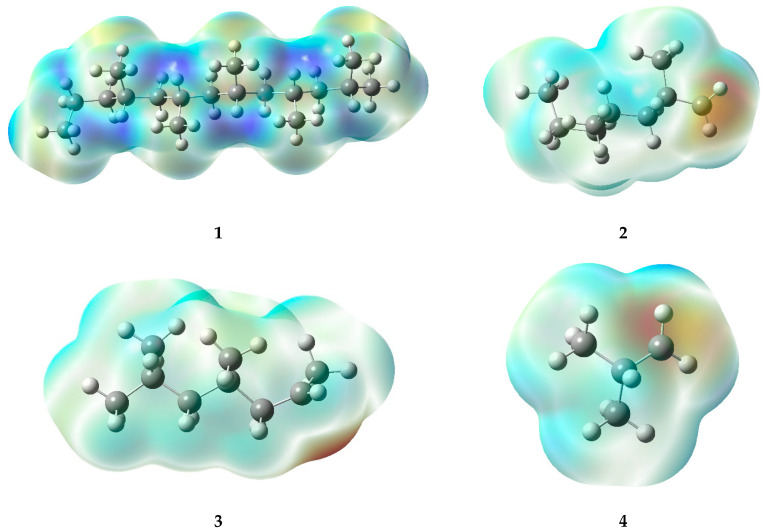

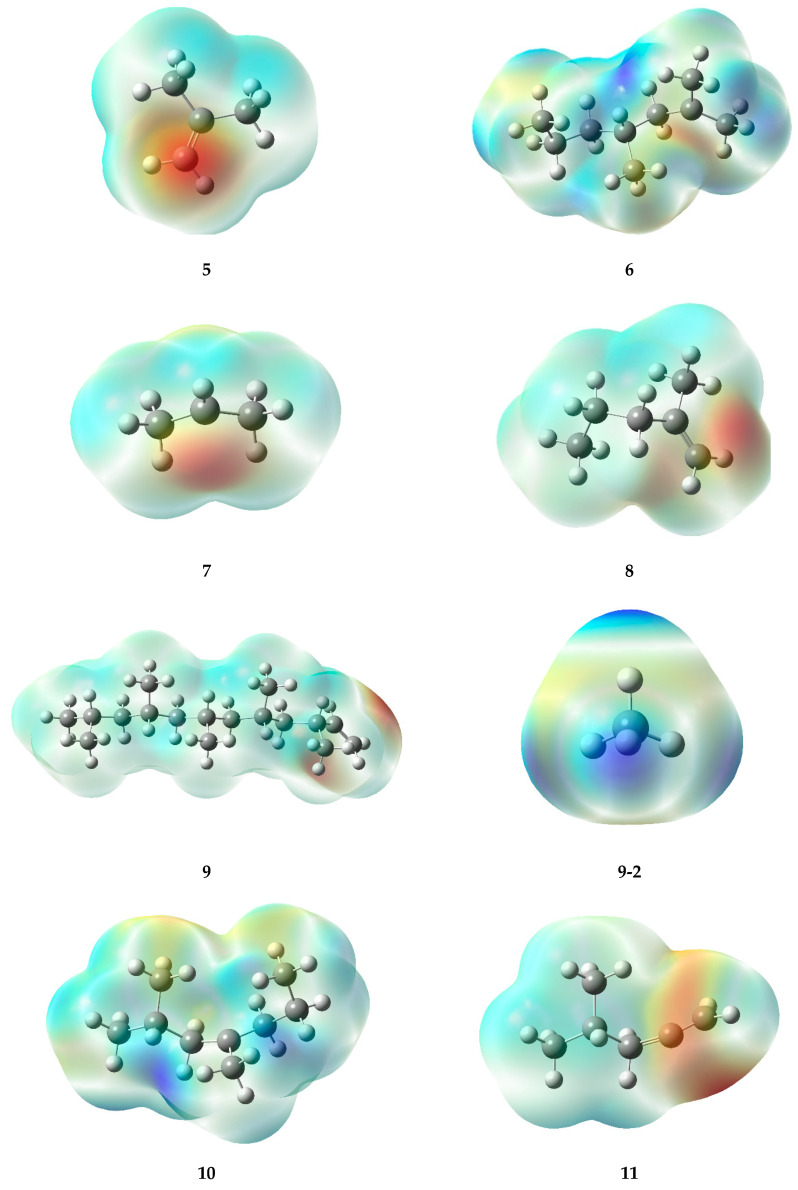

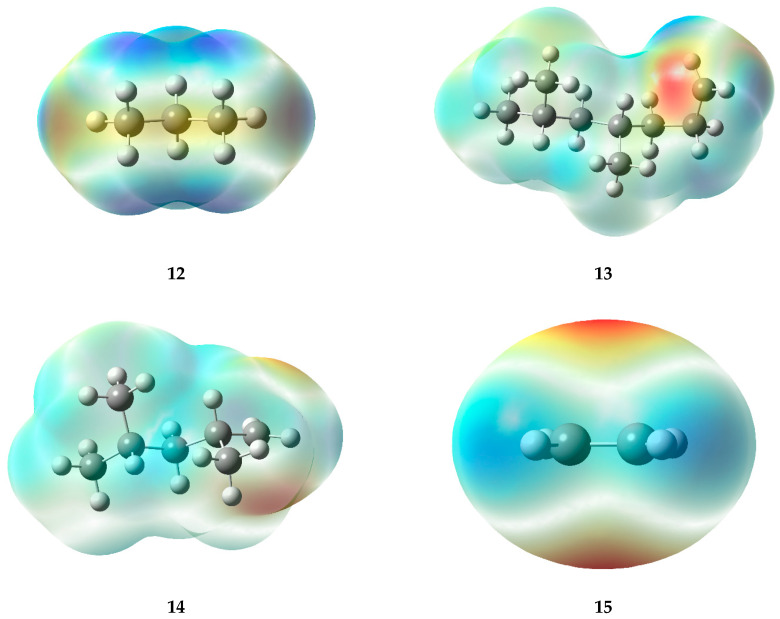
Electrostatic potential maps of the fragments generated during the PP degradation process.

The high-temperature areas located at double bonds and radicals in electrostatic potential maps (EPMs) provide consistent information about the reactivity of the molecules (Figure 7). In the case of double bonds present in specific structures (5, 8, 11, and 15), the pi electrons generated in such bonds generate a higher electron density, leading to areas of positive charge on the carbon atoms involved in the bond. Consequently, hot regions (deep red or orange) are generated in the EPM, which demonstrate a remarkable reactivity, especially toward nucleophiles or compounds with high electron density.

In contrast, radicals, which are characterized by the loss of an electron, also display warm-toned areas in the graphs. The lack of electrons causes the formation of a type of charge known as a “hole,” which can result in a partial positive charge on the atom hosting the radical, rather than a negative charge. In the warm areas near ions, a large negative charge is not evident; instead, a high level of reactivity can be observed. This generates interest in compounds that can donate electrons (as nucleophiles) or in molecules with a high electron density. Within radicals, it is possible to identify warm areas that indicate the presence of active regions, which provide ideal conditions for chemical reactions, similar to those seen in double bonds.

## 4. Conclusions

This study demonstrates that a 10 wt % HMOR catalyst is optimal for the thermocatalytic pyrolysis of both polypropylene (PP) and high-density polyethylene (HDPE). Under the selected conditions (300 °C, catalyst-to-polymer ratio of 10%), HMOR afforded the highest liquid-oil yields of 60.8% for PP and 87.3% for HDPE, while simultaneously delivering superior fuel properties (PCS of 44.2 MJ/kg and RON 97.5 for PP-derived liquids; PCS of 43.7 MJ/kg and RON 115.2 for HDPE-derived liquids). Above 400 °C, the shift toward gas production (exceeding 50 wt% at 441 °C) underscores the pivotal role of temperature in steering product selectivity.

By integrating experimental thermocatalytic trials, Aspen Plus process simulations, and density functional theory (DFT) calculations, this work fills a critical gap in plastic-waste valorization research: the lack of a unified, multiscale framework that connects catalyst acidity and molecular-scale reaction mechanisms to process-scale performance metrics. The validated simulation model (deviations ≤ 20% relative to experimental data) serves as a predictive tool for scaling up, enabling practitioners to forecast product distributions and energy outputs across a broad range of operating parameters without exhaustive empirical testing.

Beyond confirming the superior performance of HMOR, which exhibited the highest acidity among the tested catalysts (425 mV, 2.3 meq/g) and outperformed copper-modified analogues, our mechanistic insights reveal how C–C bond-cleavage pathways evolve with temperature. DFT-derived energy profiles and molecular electrostatic potential maps elucidate the thermodynamics and kinetics underpinning radical formation at 500–550 °C, offering guidance for future catalyst design to further suppress unwanted gaseous by-products and enhance liquid yields.

## Figures and Tables

**Table 1:** Characteristics of the HMOR catalyst.

Catalyst	Micropore Dimension (nm)	BET Area (cm^3^/g)	Si/Al Ratio	Trade Name	Brand
HMOR	0.65 × 0.70	561	6.3	H-Mordenita	Sigma Aldrich (St. Louis, MO, USA)

**Table 2:** Table of experimental conditions for thermal and catalytic pyrolysis, detailing temperature, catalyst–raw material ratio, and reactor type.

#	Pyrolysis Type	Category	Temperature	Catalyst–Raw Material Ratio (% *m*/*m*)	Reactor Type
1	Cat	HMOR	400	5	Quartz pyrolyzer
2	Cat	CuCl_2_-HMOR	400	5	
3	Cat	CuSO_4_-HMOR	400	5	
4	Cat	CuNO_3_-HMOR	400	5	
1	Cat	HMOR	450	15	
2	Cat	CuCl_2_-HMOR	450	15	
3	Cat	CuSO_4_-HMOR	450	15	
4	Cat	CuNO_3_-HMOR	450	15	
1	Cat	HMOR	500	30	
2	Cat	CuCl_2_-HMOR	500	30	
3	Cat	CuSO_4_-HMOR	500	30	
4	Cat	CuNO_3_-HMOR	500	30	

**Table 3:** Conditions used to carry out the thermal and catalytic pyrolysis of polypropylene.

No. of the Experiment	Catalyst–Raw Material Ratio (% *m*/*m*)	Process Temperature (°C)
1	10	158
2	5	200
3	15	200
4	10	300
5	10	300
6	10	300
7	10	300
8	10	300
9	2.93	300
10	17.07	300
11	5	400
12	15	400
13	10	441

**Table 4:** Aspen Plus models were used for each unit operation of the pyrolysis process.

Equipment Name	Aspen Plus Model	Remarks
DESCOM	RYIELD	Reactor for decomposing plastics at isothermal temperatures (440 °C or 550 °C) and 1 atm pressure
INTERCAM	HEATER	Gas heat exchanger up to 29.8 °C
SEP-1	SSPLIT	Filter for the recovery of particulate material
SEP-2	FLASH	Separate liquids from non-condensable gases
REACTOR	RGIBBS (thermodynamic), RBATCH (kinetic)	Pyrolytic reactor (400 °C or 550 °C and isobaric)

**Table 5:** Next and final analysis for PET and HDPE.

Characterization	No. of Replicas	PP	HDPE
Moisture, %	3	0	0.011
Fixed Carbon, %	3	0	0.00
Volatile matter, %	3	99	96.79
Ash, %	3	1	3.14
C, %	3	84	82.59
H, %	3	14	13.29
O, %	3	2	0.38
N, %	3	0	0.00
S, %	3	0	0.52

**Table 6:** Analysis of the maximum acidity values and the quantity of acid sites present in the HMOR catalyst and its modifications.

Catalyst	Acidity (mV)	Number of Acid Sites (meq/g)
HMOR	425	2.3
CuCl_2_-HMOR	290	2.0
CuSO_4_-HMOR	250	1.8
CuNO_3_-HMOR	233	1.8

**Table 7:** Results of the pyrolysis experiments, detailing the catalyst-to-feedstock ratio, process temperature, yield of liquid formed, performance prediction, percentage of carbon in the sample, and rate of gas generated.

Experiment No.	Ratio of Catalyst to Raw Material (% *w*/*w*)	Process Temperature (°C)	Percentage of Liquid Yield Formed	Performance Prediction	Percentage of Carbon in the Sample (by Mass)	Mass Percentage of Gas
1	10	158	0	10	98.80	1.34
2	5	200	20.74	20.80	55.85	23.62
3	15	200	9.50	9.58	69.60	21.40
4	10	300	60.80	60.60	14.72	25.0
5	10	300	60.20	60.40	15.40	24.80
6	10	300	60.20	60.40	15.30	24.60
7	10	300	60.40	60.42	15.0	24.79
8	10	300	60.75	60.42	14.40	25.02
9	2.93	300	43.90	44.05	6.24	50.35
10	17.07	300	44.80	44.72	16.45	39.04
11	5	400	40.15	39.85	7.33	52.75
12	15	400	52.25	52.00	19.00	29.30
13	10	441	38.10	38.42	10.25	52.30

**Table 8:** CCD (Central Composite Design) array with efficient extraction of liquid oil, both in experimental tests and in projections for recycled HDPE plastics.

Experiment No.	Ratio of Catalyst to Raw Material (% *w*/*w*)	Process Temperature (°C)	Percentage of Liquid Yield Formed	Performance Prediction	Percentage of Carbon in the Sample (by Mass)	Mass Percentage of Gas	Strong Performance (%)
1	10	158	0	10	98.80	1.34	98.66
2	5	200	20.74	20.80	55.85	23.62	55.64
3	15	200	9.50	9.58	69.60	21.40	69.10
4	10	300	60.80	60.60	14.72	25.0	14.20
5	10	300	60.20	60.40	15.40	24.80	15.00
6	10	300	60.20	60.40	15.30	24.60	15.20
7	10	300	60.40	60.42	15.0	24.79	14.81
8	10	300	60.75	60.42	14.40	25.02	14.23
9	2.93	300	43.90	44.05	6.24	50.35	5.75
10	17.07	300	44.80	44.72	16.45	39.04	16.16
11	5	400	40.15	39.85	7.33	52.75	7.10
12	15	400	52.25	52.00	19.00	29.30	18.45
13	10	441	38.10	38.42	10.25	52.30	9.60

**Table 9:** Simulation results for the quality of liquids obtained by pyrolysis of plastic waste.

	PP		HDPE		Gasoline	Diesel
	L	S	L	S		
PCS (Mj/Kg)	40.6	44.2	40.2	38.8	42.1	42.7
Minimum octane rating (MON)	87.4	81.7	85.5	82.5	82–91	-
Minimum octane rating (ron)	97.5	102.4	95.6	102.8	92–100	-
Antiknock index	92.5	92.2	90.5	92.7	81.5	-
Density @15 °C (g/cm^3^)	0.88	0.96	0.92	0.85	0.82	0.85

**Table 10:** Distribution of compositions based on simulation data.

PP		HDPE		RSD
Compound	% Weight (Average 5 Replicates)	Compound	% Weight (Average 5 Replicates)	
C_12_H_10_	10	C_7_H_8_	22.2	<3
C_11_H_10_	9.5	C_8_H_10_	14.3	<3
C_14_H_10_	8.1	C_14_H_30_	12.2	<3
C_8_H_10_	4.2	C_15_H_32_	6.5	<3
C_8_H_10_C	4.3	C_16_H_34_	4.5	<3
C_7_H_8_	2.5	C_6_H_6_	3.8	<3
C_9_H_12_	1.3	C_9_H_18_	3.4	<3
C_6_H_6_	1.28	C_11_H_24_	3	<3
Others	59.5	Others	30.1	<3
CH_4_	20	CH_4_	5	<3
C_2_H_6_	19.5	C_2_H_4_	4.6	<3
C_3_H_6_	3.2	H_2_	1.5	<3
C_2_H_6_	3.1	C_3_H_6_	1.9	<3
Others	53.6	Others	86.5	<3

**Table 11:** Results obtained from the PP reaction route at 450 °C under pressure conditions of 1 atm, 10 atm, and 20 atm. The thermodynamic properties are presented using SI units: system energy (E), Gibbs free energy (G), and enthalpy (H) are expressed in joules (J), while entropy (S) is given in kilojoules per mole (kJ/mol).

450 °C–1 atm					450 °C–10 atm				450 °C–20 atm			
**Molecule #**	E (J)	G (J)	H (J)	S (kJ/mol)	E (J)	G (J)	H (J)	S (kJ/mol)	E (J)	G (J)	H (J)	S (kJ/mol)
1	−3.09 × 10^−12^	−3.09 × 10^−12^	−3.09 × 10^−12^	1.25 × 10^6^	−3.09 × 10^−12^	−3.09 × 10^−12^	−3.09 × 10^−12^	1.23 × 10^6^	−3.09 × 10^−12^	−3.09 × 10^−15^	−3.09 × 10^−12^	1.22 × 10^6^
2	−1.54 × 10^−12^	−1.54 × 10^−15^	−1.54 × 10^−12^	7.31 × 10^5^	−1.54 × 10^−12^	−1.54 × 10^−12^	−1.54 × 10^−12^	7.12 × 10^5^	−1.54 × 10^−12^	−1.54 × 10^−12^	−1.54 × 10^−12^	7.06 × 10^5^
3	−1.54 × 10^−12^	−1.54 × 10^−15^	−1.54 × 10^−12^	7.35 × 10^5^	−1.54 × 10^−15^	−1.54 × 10^−12^	−1.54 × 10^−12^	7.16 × 10^5^	−1.54 × 10^−15^	−1.54 × 10^−12^	−1.54 × 10^−12^	7.10 × 10^5^
4	−6.87 × 10^−13^	−6.88 × 10^−13^	−6.87 × 10^−13^	4.36 × 10^5^	−6.87 × 10^−16^	−6.88 × 10^−13^	−6.87 × 10^−13^	4.17 × 10^5^	−6.87 × 10^−16^	−6.88 × 10^−13^	−6.87 × 10^−13^	4.11 × 10^5^
5	−6.85 × 10^−13^	−6.86 × 10^−13^	−6.85 × 10^−13^	4.05 × 10^5^	−6.85 × 10^−13^	−6.86 × 10^−13^	−6.85 × 10^−13^	3.86 × 10^5^	−6.85 × 10^−13^	−6.86 × 10^−13^	−6.85 × 10^−13^	3.81 × 10^5^
6	−1.54 × 10^−12^	−1.54 × 10^−12^	−1.54 × 10^−12^	7.40 × 10^5^	−1.54 × 10^−12^	−1.54 × 10^−15^	−1.54 × 10^−12^	7.21 × 10^5^	−1.54 × 10^−12^	−1.54 × 10^−12^	−1.54 × 10^−12^	7.15 × 10^5^
7	−5.16 × 10^−13^	−5.17 × 10^−13^	−5.16 × 10^−13^	3.87 × 10^5^	−5.16 × 10^−13^	−5.17 × 10^−13^	−5.16 × 10^−13^	3.67 × 10^5^	−5.16 × 10^−13^	−5.17 × 10^−13^	−5.16 × 10^−13^	3.61 × 10^5^
8	−1.03 × 10^−12^	−1.03 × 10^−12^	−1.03 × 10^−12^	5.23 × 10^5^	−1.03 × 10^−12^	−1.03 × 10^−12^	−1.03 × 10^−12^	5.04 × 10^5^	−1.03 × 10^−12^	−1.03 × 10^−12^	−1.03 × 10^−12^	4.98 × 10^5^
9	−2.91 × 10^−12^	−2.91 × 10^−12^	−2.91 × 10^−12^	1.16 × 10^6^	−2.91 × 10^−12^	−2.91 × 10^−12^	−2.91 × 10^−12^	1.14 × 10^6^	−2.91 × 10^−12^	−2.91 × 10^−12^	−2.91 × 10^−12^	1.14 × 10^6^
9_2	−1.76 × 10^−13^	−1.77 × 10^−13^	−1.76 × 10^−13^	2.46 × 10^5^	−1.76 × 10^−16^	−1.77 × 10^−13^	−1.76 × 10^−13^	2.27 × 10^5^	−1.76 × 10^−16^	−1.77 × 10^−13^	−1.76 × 10^−13^	2.21 × 10^5^
10	−1.54 × 10^−12^	−1.54 × 10^−12^	−1.54 × 10^−12^	7.33 × 10^5^	−1.54 × 10^−12^	−1.54 × 10^−12^	−1.54 × 10^−12^	7.13 × 10^5^	−1.54 × 10^−12^	−1.54 × 10^−12^	−1.54 × 10^−12^	7.08 × 10^5^
11	−1.02 × 10^−12^	−1.03 × 10^−12^	−1.02 × 10^−12^	5.26 × 10^5^	−1.02 × 10^−12^	−1.03 × 10^−12^	−1.02 × 10^−12^	5.06 × 10^5^	−1.02 × 10^−12^	−1.03 × 10^−12^	−1.02 × 10^−12^	5.00 × 10^5^
12	−5.19 × 10^−13^	−5.19 × 10^−13^	−5.19 × 10^−13^	3.67 × 10^5^	−5.19 × 10^−13^	−5.19 × 10^−13^	−5.19 × 10^−13^	3.48 × 10^5^	−5.19 × 10^−13^	−5.19 × 10^−13^	−5.19 × 10^−13^	3.42 × 10^5^
13	−1.54 × 10^−12^	−1.54 × 10^−12^	−1.54 × 10^−12^	7.28 × 10^5^	−1.54 × 10^−12^	−1.54 × 10^−12^	−1.54 × 10^−12^	7.10 × 10^5^	−1.54 × 10^−12^	−1.54 × 10^−12^	−1.54 × 10^−12^	7.04 × 10^5^
14	−1.20 × 10^−12^	−1.20 × 10^−12^	−1.20 × 10^−12^	6.11 × 10^5^	−1.20 × 10^−12^	−1.20 × 10^−12^	−1.20 × 10^−12^	5.92 × 10^5^	−1.20 × 10^−12^	−1.20 × 10^−12^	−1.20 × 10^−12^	5.87 × 10^5^
15	−3.42 × 10^−13^	−3.43 × 10^−13^	−3.42 × 10^−13^	2.82 × 10^5^	−3.42 × 10^−13^	−3.43 × 10^−13^	−3.42 × 10^−13^	2.63 × 10^5^	−3.42 × 10^−13^	−3.43 × 10^−13^	−3.42 × 10^−13^	2.58 × 10^5^

**Table 12:** Results obtained from the PP reaction route at 550 °C under pressure conditions of 1 atm, 10 atm, and 20 atm. The thermodynamic properties are presented using SI units: system energy (E), Gibbs free energy (G), and enthalpy (H) are expressed in joules (J), while entropy (S) is given in kilojoules per mole (kJ/mol).

550 °C–1 atm					550 °C–10 atm				550 °C–20 atm			
Molecule #	E (J)	G (J)	H (J)	S (kJ/mol)	E (J)	G (J)	H (J)	S (kJ/mol)	E (J)	G (J)	H (J)	S (kJ/mol)
1	−3.09 × 10^−12^	−3.09 × 10^−12^	−3.09 × 10^−15^	1.36 × 10^6^	−3.09 × 10^−12^	−3.09 × 10^−12^	−3.09 × 10^−12^	1.34 × 10^6^	−3.09 × 10^−12^	−3.09 × 10^−12^	−3.09 × 10^−15^	1.33 × 10^6^
2	−1.54 × 10^−12^	−1.54 × 10^−12^	−1.54 × 10^−12^	7.85 × 10^5^	−1.54 × 10^−12^	−1.54 × 10^−12^	−1.54 × 10^−12^	7.66 × 10^5^	−1.54 × 10^−12^	−1.54 × 10^−12^	−1.54 × 10^−12^	7.60 × 10^5^
3	−1.54 × 10^−12^	−1.54 × 10^−12^	−1.54 × 10^−12^	7.89 × 10^5^	−1.54 × 10^−12^	−1.54 × 10^−12^	−1.54 × 10^−12^	7.70 × 10^5^	−1.54 × 10^−12^	−1.54 × 10^−12^	−1.54 × 10^−12^	7.64 × 10^5^
4	−6.87 × 10^−13^	−6.88 × 10^−13^	−6.87 × 10^−13^	4.61 × 10^5^	−6.87 × 10^−13^	−6.88 × 10^−13^	−6.87 × 10^−13^	4.41 × 10^5^	−6.87 × 10^−13^	−6.88 × 10^−13^	−6.87 × 10^−13^	4.36 × 10^5^
5	−6.85 × 10^−13^	−6.86 × 10^−13^	−6.85 × 10^−13^	4.28 × 10^5^	−6.85 × 10^−13^	−6.86 × 10^−13^	−6.85 × 10^−13^	4.09 × 10^5^	−6.85 × 10^−13^	−6.86 × 10^−13^	−6.85 × 10^−13^	4.03 × 10^5^
6	−1.54 × 10^−12^	−1.54 × 10^−12^	−1.54 × 10^−12^	7.95 × 10^5^	−1.54 × 10^−12^	−1.54 × 10^−12^	−1.54 × 10^−12^	7.75 × 10^5^	−1.54 × 10^−12^	−1.54 × 10^−12^	−1.54 × 10^−12^	7.69 × 10^5^
7	−5.16 × 10^−13^	−5.17 × 10^−13^	−5.16 × 10^−13^	4.05 × 10^5^	−5.16 × 10^−13^	−5.17 × 10^−13^	−5.16 × 10^−13^	3.86 × 10^5^	−5.16 × 10^−13^	−5.17 × 10^−13^	−5.16 × 10^−13^	3.80 × 10^5^
8	−1.03 × 10^−12^	−1.03 × 10^−12^	−1.03 × 10^−12^	5.58 × 10^5^	−1.03 × 10^−12^	−1.03 × 10^−12^	−1.03 × 10^−12^	5.38 × 10^5^	−1.03 × 10^−12^	−1.03 × 10^−12^	−1.03 × 10^−12^	5.33 × 10^5^
9	−2.91 × 10^−12^	−2.91 × 10^−12^	−2.91 × 10^−12^	1.26 × 10^6^	−2.91 × 10^−12^	−2.91 × 10^−12^	−2.91 × 10^−12^	1.24 × 10^6^	−2.91 × 10^−12^	−2.91 × 10^−12^	−2.91 × 10^−12^	1.24 × 10^6^
9_2	−1.76 × 10^−13^	−1.77 × 10^−13^	−1.76 × 10^−13^	2.54 × 10^5^	−1.76 × 10^−13^	−1.77 × 10^−13^	−1.76 × 10^−13^	2.35 × 10^5^	−1.76 × 10^−13^	−1.77 × 10^−13^	−1.76 × 10^−13^	2.29 × 10^5^
10	−1.54 × 10^−12^	−1.54 × 10^−12^	−1.54 × 10^−12^	7.87 × 10^5^	−1.54 × 10^−12^	−1.54 × 10^−12^	−1.54 × 10^−12^	7.68 × 10^5^	−1.54 × 10^−12^	−1.54 × 10^−12^	−1.54 × 10^−12^	7.62 × 10^5^
11	−1.02 × 10^−12^	−1.03 × 10^−12^	−1.02 × 10^−15^	5.59 × 10^5^	−1.02 × 10^−12^	−1.03 × 10^−12^	−1.02 × 10^−12^	5.39 × 10^5^	−1.02 × 10^−12^	−1.03 × 10^−12^	−1.02 × 10^−12^	5.33 × 10^5^
12	−5.19 × 10^−13^	−5.20 × 10^−13^	−5.19 × 10^−13^	3.87 × 10^5^	−5.19 × 10^−13^	−5.20 × 10^−13^	−5.19 × 10^−13^	3.67 × 10^5^	−5.19 × 10^−13^	−5.20 × 10^−13^	−5.19 × 10^−13^	3.61 × 10^5^
13	−1.54 × 10^−12^	−1.54 × 10^−15^	−1.54 × 10^−12^	7.83 × 10^5^	−1.54 × 10^−12^	−1.54 × 10^−12^	−1.54 × 10^−12^	7.64 × 10^5^	−1.54 × 10^−12^	−1.54 × 10^−12^	−1.54 × 10^−12^	7.58 × 10^5^
14	−1.20 × 10^−12^	−1.20 × 10^−12^	−1.20 × 10^−12^	6.54 × 10^5^	−1.20 × 10^−12^	−1.20 × 10^−12^	−1.20 × 10^−12^	6.35 × 10^5^	−1.20 × 10^−12^	−1.20 × 10^−12^	−1.20 × 10^−12^	6.29 × 10^5^
15	−3.42 × 10^−13^	−3.43 × 10^−16^	−3.42 × 10^−13^	2.93 × 10^5^	−3.42 × 10^−13^	−3.43 × 10^−13^	−3.42 × 10^−13^	2.74 × 10^5^	−3.42 × 10^−13^	−3.43 × 10^−13^	−3.42 × 10^−13^	2.68 × 10^5^

## Data Availability

The original contributions presented in this study are included in the article. Further inquiries can be directed to the corresponding authors.

## List of Abbreviations

PP	Polypropylene
HDPE	High-density polyethylene
DFT	Density functional theory
ReaxFF MD	Reactive molecular dynamics with the ReaxFF force field
RGIBBS	Aspen Plus equilibrium reactor
RBATCH	Aspen Plus kinetic batch reactor
R-YIELD	Aspen Plus reactor for conversion of non-routine to routine streams
HMOR	H-Mordenite zeolite
CuCl_2_-HMOR, CuSO_4_-HMOR, CuNO_3_-HMOR	HMOR modified by ion exchange with CuCl_2_, CuSO_4_ or CuNO_3_, respectively
PCS	Power-to-weight ratio
RON	Research Octane Number
MON	Motor Octane Number
PR-EOS	Peng–Robinson equation of state
NRTL	Non-Random Two-Liquid activity coefficient model
CCD	Central Composite Design
HOMO	Highest Occupied Molecular Orbital
LUMO	Lowest Unoccupied Molecular Orbital
EPM	Electrostatic potential map
TGA	Thermogravimetric analysis
VM	Volatile matter
FC	Fixed carbon
ASTM	American Society for Testing and Materials
BET	Brunauer–Emmett–Teller
GC-MS	Gas chromatography–mass spectrometry
FTIR	Fourier-transform infrared spectroscopy
C, H, N, O, S	Carbon, hydrogen, nitrogen, oxygen, and sulfur
